# Case report: primary pericardial tumour—challenges in imaging diagnosis and management

**DOI:** 10.1093/ehjcr/ytag151

**Published:** 2026-03-06

**Authors:** Nguyen Nhat Dinh Ngo

**Affiliations:** Department of Cardiology, Hoan My Saigon Hospital, 60-60A Phan Xich Long Street, Ward 1, Phu Nhuan District, Ho Chi Minh City, Vietnam

**Keywords:** Primary pericardial tumour, Pericardial effusion, Atrial fibrillation, Cardiac tamponade, Multimodal imaging, Sarcoma, Case report

## Abstract

**Background:**

Primary pericardial tumours are exceedingly rare and frequently misdiagnosed due to nonspecific clinical presentations. They may initially manifest through cardiovascular complications such as pericardial effusion, arrhythmias, or embolic events, creating significant diagnostic and management challenges.

**Case summary:**

We report a 62-year-old female with a history of dyslipidaemia who was admitted for progressive dyspnoea, mild chest pain, and palpitations. Cardiovascular evaluation revealed new-onset atrial fibrillation, large pericardial effusion, and bilateral pleural effusions. Transthoracic echocardiography and contrast-enhanced computed tomography identified a heterogeneous pericardial mass with invasive features involving the pulmonary artery, complicated by pulmonary artery obstruction. The patient developed cardiac tamponade requiring emergent pericardiocentesis, which drained 550 mL of serous fluid. Cytological analysis of the pericardial fluid suggested a malignant mesenchymal tumour, and subsequent CT-guided biopsy raised strong suspicion for a primary pericardial sarcoma, pending immunohistochemical confirmation. The patient was managed with anticoagulation, rate control for atrial fibrillation, supportive therapy, and multidisciplinary consultation, leading to a planned oncologic treatment strategy.

**Conclusion:**

This case highlights the pivotal role of cardiology in recognizing occult malignancy presenting with acute cardiovascular manifestations, the diagnostic complexity of primary pericardial tumours, and the importance of multimodal imaging, histopathology, and multidisciplinary collaboration in emergency and long-term management.

Learning points

1. Primary pericardial tumours are rare and may initially present with atypical cardiovascular manifestations such as pericardial effusion, arrhythmias, or thromboembolic events.
2. Multimodal imaging, including echocardiography, computed tomography, and cardiac magnetic resonance, is essential to determine tumour origin, extent, and haemodynamic impact.
3. Histological confirmation by biopsy is mandatory, and optimal management requires a multidisciplinary approach integrating surgery and oncological therapies.

## Introduction

Most primary pericardial tumours are benign (lipoma, fibroma, teratoma, vascular, schwannoma) but can still cause complications by compressing nearby structures or inducing arrhythmias.^1–3^ A minority are malignant, mainly sarcomas (angiosarcoma, rhabdomyosarcoma), with rarer types like malignant fibrous histiocytoma and lymphoma.^4–6^ The pericardium may also be secondarily involved by metastases, commonly from melanoma, lung, breast, renal, or oesophageal cancers.^1^

We report a rare case of suspected primary pericardial sarcoma presenting with effusion, atrial fibrillation, and pulmonary embolism, with imaging revealing pulmonary artery invasion. This case highlights the diagnostic challenges and underscores the importance of multimodal imaging, timely drainage, histopathological confirmation, and early multidisciplinary management.^7–10^ Such uncommon presentations are rarely reported and provide valuable insight into the clinical course and treatment of pericardial sarcoma, especially when surgery is not feasible due to invasion or comorbidities.

## Summary figure

**Table:** 

Date (2025)	Event/Findings	Intervention/Outcome
1 July	Admission with dyspnoea, chest pain, palpitations; AF with HR 116 bpm; pericardial & pleural effusion	Supportive care
2 July	Echocardiography: moderate pericardial effusion; preserved LV function	Supportive care
3 July	Chest CT: pericardial mass 32 × 40 mm compressing pulmonary artery; pulmonary embolism; pleural effusion	Supportive care
4 July	Pericardiocentesis: 550 mL drained; cytology: mesenchymal tumour	Symptom relief
5 July	CT-guided biopsy: suspicious sarcoma (pending IHC)	Diagnosis confirmed
6–7 July	Started anticoagulation (Enoxaparin → Rivaroxaban), bisoprolol, digoxin, furosemide, atorvastatin, esomeprazole	Stable
Ongoing	Multidisciplinary Oncology Consultation; Chemotherapy (Ifosfamide–Doxorubicin ± Radiotherapy) Planned	Prognosis Guarded

## Clinical case

A 62-year-old woman with severe hypertriglyceridaemia and a history of dyslipidaemia was admitted to the hospital with progressive shortness of breath, mild chest pain, and palpitations. She had no history of chronic illnesses, previous hospitalizations, regular medication use, or relevant family history. Her symptoms had developed gradually over several days, without any recent infection or trauma. She denied tobacco use, alcohol consumption, or recreational drug use. The patient had a body mass index of 24.1 kg/m^2^ and reported no recent weight loss.

### Physical examination

On physical examination, the patient was tachycardic with an irregular heart rate of 116 beats per minute and a blood pressure of 110/70 mmHg. The respiratory rate was 22 breaths per minute, with an oxygen saturation of 95% on room air. Pulmonary examination revealed dullness to percussion over the lower third of the right lung, associated with decreased breath sounds. Cardiac auscultation demonstrated an irregular tachycardia with distant heart sounds. Abdominal examination was notable for epigastric tenderness.

### Paraclinical investigations

#### Laboratory investigations

Initial laboratory testing demonstrated leukocytosis and elevated inflammatory markers, with a peak white blood cell count of 16.4 × 10^9^/L and a C-reactive protein level of 155 mg/L, which gradually decreased during hospitalization (*Table 1*). Haemoglobin levels were mildly reduced, while platelet counts remained within the normal range. Cardiac biomarkers, including troponin I and NT-proBNP, were within normal limits. D-dimer levels were mildly elevated. Renal and liver function tests were largely unremarkable. Arterial blood gas analysis showed mild hypocapnia with borderline hypoxaemia.

**Table 1:** Laboratory investigations

Test	Result				Reference range
	1/7/2025	3/7/2025	5/7/2025	7/7/2025	
WBC	16.4	15.8	13.1	12.0	3.68–12.2 × 10E9/L
Hgb	111	106	101	102	111.9–155.6 g/L
PLT	305	297	267	265	150–350 × 10E9/L
CRP	155	101	94	88	
Procalcitonin (PCT)	0.07				<0.5
D-dimer	597				<500 ng/mL
NT-proBNP	25				<125 pg/mL
Troponin I	<10	<10	<10	<10	<15.6 ng/L
LDL-c	1.14				<1.7 mmol/L
Triglyceride	3.24				<3.367 mmol/L
Creatinine	56	67	54	60	44.2–106.1 umol/L
Glucose	6.99				4.6–6.4 mmol/L
AST	52				0–55 U/L
ALT	41.55				5–34 U/L
Sodium (Na)	138.28				136–145 mmol/L
Potassium (K)	3.66				3.5–5.1 mmol/L
Calcium (Ca)	2.02				2.24–2.7 mmol/L
TSH	2.0297				0.35–4.94 uIU/mL
Free T4 II	17.8925				9.01–19.05 pmolL
LDH	248				140–280 U/L
ESR	68				<30 mm/h
**Arterial blood gas (ABG) analysis:**					
Temperature	37				degrees Celsius
FiO_2_	0.37				
PCO_2_	31.8				35–45 mmHg
PO_2_	76.8				80–105 mmHg
SO_2_(c)	95.9				95–98%

#### Fluid analysis and electrocardiography

Analysis of the pericardial fluid revealed a lactate dehydrogenase level of 729 U/L. Electrocardiography demonstrated atrial fibrillation with a rapid ventricular response, without electrocardiographic signs of myocardial ischaemia.

#### Chest radiography

**Figure 1:**
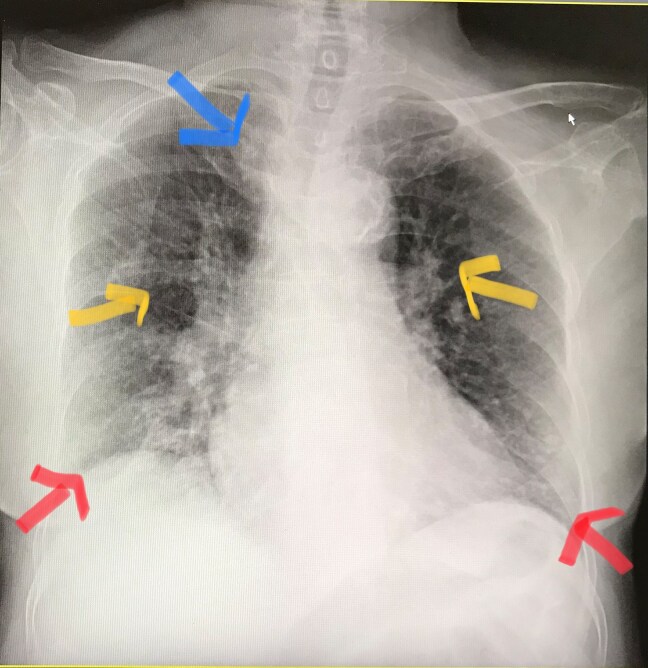
Chest radiograph demonstrating bilateral pleural effusions, interstitial lung involvement, and consolidation in the right upper lobe.

Chest radiography revealed large-volume bilateral pleural effusions, consolidation in the right upper lung zone, and diffuse interstitial abnormalities (*Figure 1*).

#### Echocardiography

Transthoracic echocardiography performed on admission demonstrated a large pericardial effusion without evidence of cardiac tamponade. Left ventricular systolic function was preserved, with an estimated ejection fraction of 66%, and no intracardiac thrombus was identified.

Repeat echocardiography performed one day later showed progression to cardiac tamponade. Given the patient’s haemodynamic instability, emergent pericardiocentesis was undertaken to relieve pericardial pressure and stabilize the patient.

**Figure 2:**
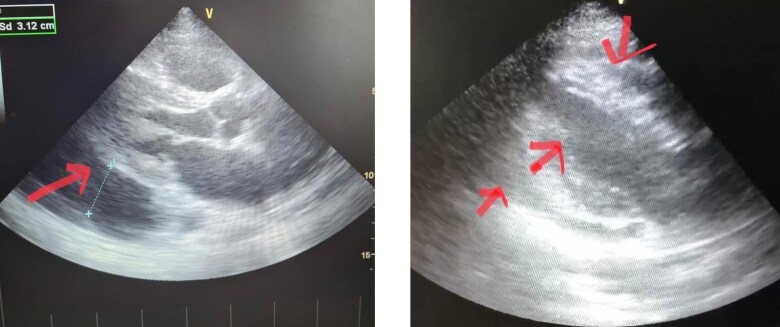
Transthoracic echocardiogram before pericardiocentesis demonstrating a large pericardial effusion.

**Figure 3:**
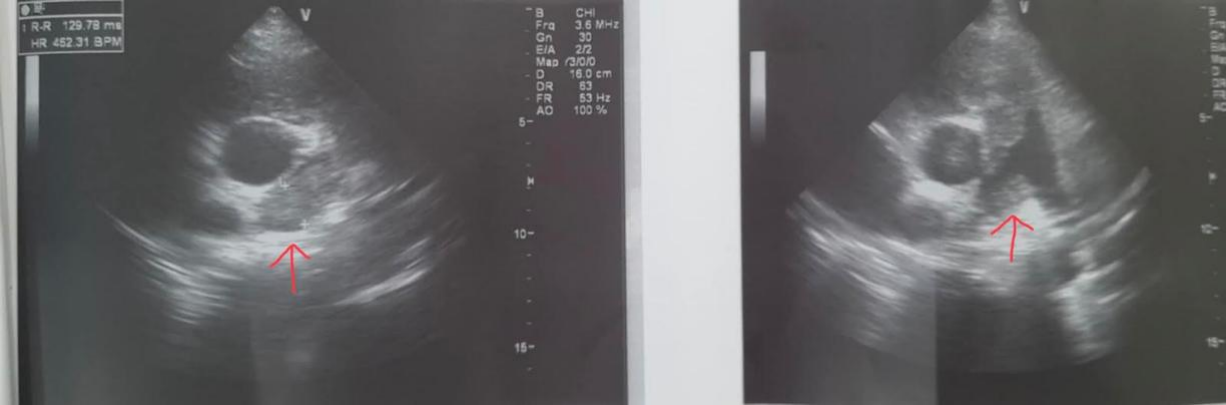
Transthoracic echocardiogram after pericardial and pleural fluid drainage showing complete resolution of the effusion.

Following pericardiocentesis, pleural fluid aspiration, and pericardial drainage, echocardiographic assessment confirmed complete resolution of the pericardial and pleural effusions. A heterogeneous mass with ill-defined invasive margins, inseparable from the pulmonary artery wall, was subsequently visualized (*Figures 2* and *3*).

#### Contrast-enhanced chest computed tomography

**Figure 4:**
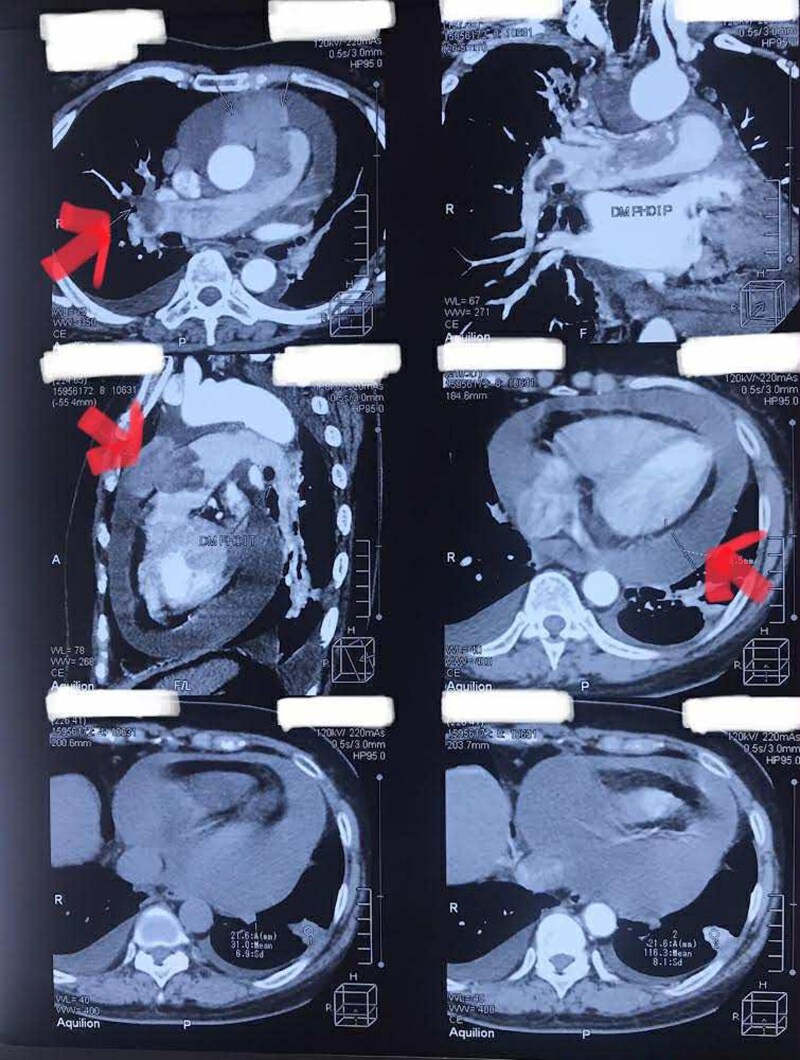
Contrast-enhanced chest computed tomography demonstrating a pericardial mass invading the pulmonary artery, with an associated intraluminal thrombus.

Contrast-enhanced chest computed tomography revealed a thrombus measuring approximately 30 mm within the main pulmonary artery, causing approximately 70% luminal narrowing. Additional findings included 70% stenosis of the right pulmonary artery, occlusion of the right upper lobar artery, and 80% stenosis of the right lower lobar artery. A moderate pericardial effusion and a pericardial mass measuring 32 × 40 mm were also identified. The right upper lobe showed fibrotic collapse with adjacent bronchiectasis. An incidental 18-mm simple cyst was noted in segment VIII of the right hepatic lobe (*Figure 4*).

#### Additional investigations

Further investigations, including abdominal and thyroid ultrasonography, contrast-enhanced multislice computed tomography of the abdomen and pelvis, gastroscopy, and colonoscopy, revealed no abnormalities, with no evidence of malignancy at other sites.

### Diagnosis

The patient was diagnosed with a suspected primary pericardial malignant mesenchymal tumour, highly suggestive of sarcoma, with invasive involvement of the main pulmonary artery. The tumour was complicated by a large pericardial effusion leading to cardiac tamponade, as well as bilateral pleural effusions. In addition, the patient had new-onset atrial fibrillation, with a CHA_2_DS_2_-VASc score of 0 and a HAS-BLED score of 0, and pulmonary artery obstruction considered to be multifactorial, including thromboembolism and possible tumour invasion. Relevant comorbidities included dyslipidaemia and gastroesophageal reflux disease.

### Treatment

#### Emergency phase

Given the presence of cardiac tamponade, emergent pericardiocentesis was performed, resulting in the drainage of 550 mL of yellowish serous fluid, without evidence of purulence. Cytological analysis of the pericardial fluid suggested a mesenchymal tumour. Following confirmation of pulmonary embolism on computed tomography pulmonary angiography, therapeutic anticoagulation was initiated with enoxaparin at a dose of 1 mg/kg twice daily for 7 days, subsequently transitioned to rivaroxaban 20 mg once daily.

Rate control for atrial fibrillation was achieved using bisoprolol 2.5 mg once daily in combination with digoxin 0.125 mg once daily. Intravenous diuretic therapy with furosemide (20 mg three times daily) was administered to manage volume overload. Additional medical therapy included atorvastatin 40 mg once daily for lipid management and esomeprazole 40 mg once daily for gastroesophageal reflux disease.

#### Histopathological diagnosis

A computed tomography-guided biopsy of the pericardial mass was performed. Histopathological examination revealed features consistent with a malignant mesenchymal tumour, raising strong suspicion for a primary pericardial sarcoma. Definitive tumour classification required immunohistochemical analysis, which was pending at the time of reporting.

#### Subsequent management and treatment plan

The case was discussed in a multidisciplinary team meeting involving cardiology, oncology, and pulmonology specialists. Based on the presumptive diagnosis, initiation of combination chemotherapy with ifosfamide and doxorubicin was planned, pending final immunohistochemical confirmation. Treatment response was scheduled to be reassessed after three cycles of chemotherapy, with consideration of radiotherapy in the presence of residual tumour on follow-up imaging. Serial transthoracic echocardiography was planned on a weekly basis to monitor for recurrence of pericardial effusion.

## Follow-up and outcomes

Following pericardial and pleural fluid drainage, both effusions resolved, with no evidence of recurrence on subsequent imaging. The patient remained haemodynamically stable, and her clinical condition improved, allowing for hospital discharge in a stable state. Weekly transthoracic echocardiographic follow-up was planned to monitor for recurrent pericardial effusion. The overall prognosis remained guarded, pending definitive histopathological confirmation and assessment of response to planned chemotherapy.

## Discussion

This case illustrates a rare presentation of a suspected primary pericardial sarcoma manifesting through acute cardiovascular complications, including large pericardial effusion with cardiac tamponade, new-onset atrial fibrillation, and pulmonary artery obstruction. The patient initially presented with nonspecific cardiopulmonary symptoms, emphasizing the pivotal role of cardiologists in recognizing occult malignancy presenting as a cardiovascular emergency.

Primary pericardial tumours are exceedingly rare and are most often detected when complications arise from pericardial effusion, myocardial or vascular compression, or arrhythmias.^1,2^ Among malignant pericardial tumours, sarcomas represent a particularly aggressive subgroup, often characterized by invasive growth patterns involving adjacent cardiac structures and major vessels.^4,11^ In this case, the presence of a heterogeneous pericardial mass with pulmonary artery invasion strongly favoured a sarcomatous origin.

Differential diagnoses of malignant pericardial tumours include primary pericardial mesothelioma and primary cardiac lymphoma. Mesothelioma, although rare, typically presents with diffuse pericardial thickening and recurrent effusions, whereas focal mass formation with vascular invasion, as observed in this patient, is more characteristic of sarcoma.^12,13^ Lymphoma usually occurs in immunocompromised patients and is often associated with systemic lymphadenopathy, which was absent in this case.

The coexistence of atrial fibrillation and pulmonary artery obstruction further supported a malignant aetiology. Atrial fibrillation may result from atrial compression or pericardial inflammation, while pulmonary artery obstruction was considered multifactorial, including true thromboembolism related to malignancy-associated hypercoagulability (Trousseau’s syndrome) and possible direct tumour invasion. Anticoagulation was therefore primarily indicated for pulmonary embolism rather than stroke prevention in atrial fibrillation.

From a diagnostic perspective, this case highlights the limitations of clinical presentation alone. Echocardiography remains the first-line modality for detecting pericardial effusion and assessing haemodynamic compromise, while computed tomography and cardiac magnetic resonance imaging play a crucial role in characterizing pericardial masses, evaluating invasiveness, and guiding biopsy.^3,6^ Histopathological confirmation remains the diagnostic gold standard, although definitive classification may be delayed due to clinical instability or limited tissue sampling.

Management of malignant pericardial tumours is challenging and requires early multidisciplinary collaboration. Surgical resection is often precluded by extensive local invasion, as in this case, making systemic chemotherapy the primary therapeutic option. Despite advances in multimodal therapy, prognosis remains poor, underscoring the importance of early recognition and coordinated cardio-oncologic care.^9,10^

## Conclusion

Primary pericardial tumours are extremely rare, diagnostically challenging, and frequently detected at an advanced stage.^1–3^ The combination of pericardial effusion, arrhythmia, and pulmonary artery obstruction should raise strong suspicion for an underlying malignant pericardial process. Multimodal imaging and histopathological evaluation are essential for diagnosis, and early multidisciplinary management is critical to optimize outcomes, even in the setting of an aggressive disease such as suspected pericardial sarcoma.^5,6^

## Supplementary Material

ytag151_Supplementary_Data

## Data Availability

The data underlying this case report are available from the corresponding author on reasonable request.

## Lead author biography

Dr Ngo Nguyen Nhat Dinh is an internist specializing in cardiology at Hoan My Saigon Hospital. She graduated from the University of Medicine and Pharmacy at Ho Chi Minh City and has over 4 years of experience in cardiology. Dr Dinh focuses on evidence-based management of cardiovascular diseases. She has three academic publications, including two national conference presentations and one national journal article. Highlights include a keynote lecture on stem cell therapy at the National Atherosclerosis Conference, a study on HFNC at the Hanoi Cardiology Conference, and a case report on severe hypertriglyceridaemia in the *Vietnam Journal of Endocrinology and Diabetes*.

## Supplementary material

**Consent:** Written informed consent was obtained from the patient for publication of this case report and images.

## Patient perspective

The patient reported relief of symptoms after drainage and was reassured by the multidisciplinary treatment plan.
